# Supplementary material for: Prospective multicentre head-to-head validation of host blood transcriptomic biomarkers for pulmonary tuberculosis by real-time PCR
Source: Commun Med (Lond). 2022 Mar 10;2:26. doi: 10.1038/s43856-022-00086-8 (PMC8954216; doi:10.1038/s43856-022-00086-8)
Supplement: Supplementary file 1 — Supplementary Information [file 43856_2022_86_MOESM1_ESM.pdf]

Prospective multicentre head-to-head validation of host blood transcriptomic biomarkers for pulmonary tuberculosis by real-time PCR

Simon C. Mendelsohn, Stanley Kimbung Mbandi, Andrew Fiore-Gartland, Adam Penn-Nicholson, Munyaradzi Musvosvi, Humphrey Mulenga, Michelle Fisher, Katie Hadley, Mzwandile Erasmus, Onke Nombida, Michèle Tameris, Gerhard Walzl, Kogieleum Naidoo, Gavin Churchyard, Mark Hatherill, and Thomas J. Scriba

SUPPLEMENTARY INFORMATION

Contents

Supplementary Notes 1 ____ 2
Supplementary Notes 2 ____ 5
Supplementary Figures ____ 6
Supplementary References ____ 21

Supplementary Notes 1

The CORTIS Study Team

Centre for the AIDS Programme of Research in South Africa (CAPRISA), and MRC-CAPRISA HIV-TB Pathogenesis and Treatment Research Unit, Doris Duke Medical Research Institute, University of KwaZulu-Natal, Durban, South Africa.
Bianca Bande
Thilagavathy Chinappa
Cara-Mia Corris
Thobelani Cwele
Celaphiwe Dlamini
Dhineshree Govender
Goodness Khanyisile Gumede
Nonhlanhla Zanele Elsie Gwamanda
Senzo Halti
Razia Hassan-Moosa
Senzo Ralph Hlathi
Zandile Patrica Jali
Lungile Khanyile
Bhavna Maharaj
Jabulisiwe Lethabo Maphanga
Nonhle Bridgette Maphanga
Siyabonga Mbatha
Atika Moosa
Nompumelelo Ngcobo
Ntombozuko Gloria Ntanjana
Sphelele Simo Nzimande
Nesri Padayatchi
Dirhona Ramjit
Thandiwe Yvonne Shezi
Zibuyile Phindile Penlee Sing
Chandrapharbha Singh
Philile Thembela
Londiwe Zaca
Mbali Ignatia Zulu

Department of Molecular Microbiology, Washington University in St. Louis, St. Louis, MO, USA
Shabaana A. Khader

Division of Medical Microbiology, Department of Pathology, University of Cape Town, Cape Town, South Africa
Slindile Mbhele
Mark P. Nicol
Judi van Heerden

DST/NRF Centre of Excellence for Biomedical TB Research and SAMRC Centre for TB Research, Division of Molecular Biology and Human Genetics, Department of Biomedical Sciences, Faculty of Medicine and Health Sciences, Stellenbosch University, Cape Town, South Africa.
Petri Ahlers
Roslyn Beukes
Maria Didloff
Marika Flinn
Bernadine Fransman
Andriëtte Hiemstra
Justine Khoury
Belinda Kriel
Jaftha Kruger
Andre Loxton
Elizna Maasdorp
Stephanus T. Malherbe
Onesisa Mpofu
Mpho Mtlali
Liesel Muller
Bronwyn Smith
Dorothy Solomons
Kim Stanley
Susanne Tonsing
Khayalethu Toto
Ayanda Tsamane
Susanne Tönsing

South African Tuberculosis Vaccine Initiative, Institute of Infectious Disease and Molecular Medicine, Division of Immunology, Department of Pathology, University of Cape Town, South Africa.
Charmaine Abrahams
Hadn Africa
Denis Arendsen
Nomfuneko Cynthia Batyi
Nicole Bilek
Natasja Botes
Samentra Braaf
Sivuyile Buhlungu
Alida Carstens
Balie Carstens
Nompumelelo Cetywayo
Yolundi Cloete
Lorraine Coetzee
Alessandro Companie
Ilse Davids
Marwou de Kock
Bongani Diamond
Palesa Dolo
Margareth Erasmus
Juanita Ferreira
Christal Ferus
Elizabeth Filander
Hennie Geldenhuys
Diann Gempies
Yolande Gregg
Rieyaat Hassiem
Roxane Herling
Yulandi Herselman
Chris Hikuam
Henry Issel
Ruwiyda Jansen
Lungisa Jaxa
Fabio Julies
Fazlin Kafaar
Masooda Kaskar
Sophie Keffers
Xoliswa Kelepu

Gloria Khomba
Sandra Kruger
Sunelza Lakay
Thelma Leopeng
Angelique Kany Kany Luabeya
Simbarashe Mabwe
Lauren Mactavie
Nomsitho Magawu
Lebohgang Makhete
Lebohang Makhethe
Faheema Meyer
Miriam Moses
Boitumelo Mosito
Angelique Mouton
Julia Noble
Nambitha Nqakala
Fajwa Opperman
Christel Petersen
Patiswa Plaatjie
Abe Pretorius
Rodney Raphela
Frances Ratangee
Maigan Ratangee
Susan Rossouw
Elisma Schoeman
Constance Schreuder
Alison September
Cashwin September
Justin Shenje
Marcia Steyn
Sonia Stryers
Liticia Swanepoel
Anne Swarts
Asma Toefy
Petrus Tyambetyu
Habibullah Valley
Linda van der Merwe
Elma van Rooyen
Ashley Veldsman
Helen Veldtsman
Kelvin Vollenhoven
Elaine Zimri

TB Modelling Group, TB Centre, Centre for Mathematical Modelling of Infectious Diseases, Department of Infectious Disease Epidemiology, London School of Hygiene & Tropical Medicine, London, United Kingdom.
Tom Sumner
Richard G. White

The Aurum Institute, Johannesburg, Gauteng, South Africa.
Aurum Klerksdorp Site
Tebogo Badimo
Kagiso Baepanye
Kesenogile Edna Baepanye
Tshepiso Baepanye
Ken Clarke
Marelize Collignon
Audrey Lebohang Dhlamini
Audrey Dlamini
Candice Eyre
Tebogo Feni
Moogo Fikizolo
Phinda Galane
Alia Gangat
Thelma Goliath
Craig Innes
Bonita Janse van Rensburg
Elba Janse van Rensburg
Olebogeng Jonkane
Boitumelo Sophy Kekana
Gomotsegang Virginia Khobedi
Marietjie King
Adrianne Kock
Ndlela Israel Kunene
Aneesa Lakhi
Nondumiso Langa
Hildah Ledwaba
Marillyn Luphoko
Immaculate Mabasa
Tshegofatso Dorah Mabe
Nkosinathi Charles Mabuza
Molly Majola
Mantai Makhetha
Blossom Makhubalo
Mpho Makoanyane
Vernon Malay
Shirley Malefo-Grootboom
Juanita Market
Selvy Matshego
Lungile Mbata
Nontsikelelo Mbipa
John Mdlulu
Tsiamo Mmotsa
Karabo Moche
Sylvester Modipa
Joseph Panie Moloko
Kabelo Molosi
Samuel Mopati
Palesa Moswegu
Primrose Mothaga
Dorothy Muller
Grace Nchwe
Nhlamulo Ndlovu
Maryna Nel
Lindiwe Nhlangulela
Tanya Nielson
Bantubonke Bertrum Ntamo
Lawerence Ntoahae
Tedrius Ntshauba
Thandiwe Papalagae
Pedro Pinho
Pearl Nomsa Sanyaka
Sharfuddin Sayed
Letlhogonolo Seabela
Raesibe Agnes Pearl Selepe
Melissa Neo Senne
Moeti Serake
Naydene Slabbert
Constance Takavamanya
Marthinette Taljaard
Maria Thlapi

Mugwena Thompo
Vincent Tshikovhi
Lebogang Isaac Tswaile
Amanda van Aswegen
Marietjie Zietsman

Aurum Rustenburg Site
Laudicia Tshenolo Bontsi
Obakeng Peter Booi
Mari Cathrin Botha
William Brumskine
Selemeng Matseliso Carol
Kgomotso Violet Chauke
Mooketsi Theophillius Cwaile
Isabella Johanna Davies
Emilia De Klerk
Blanchard Mbay Iyemosolo
James Michael Jeleni
Christian Mabika Kasongo
Sebaetseng Jeanette Kekana
Lucky Sipho Khoza
Gloria Keitumetse Kolobe
Lerato Julia Lekagane
Sheiley Christina Lekotloane
Ilze Jeanette Louw
Sarah Teboso Lusale
Perfect Tiisetso Maatjie
Kamogelo Fortunate Mabena
Johanna Thapelo Madikwe
Octavia Mahkosazana Madikwe
Rapontwana Letlhogonolo Maebana
Malobisa Sylvester Magwasha
Vutlhari-I-Vunhenha Fairlord Manzini
Isholedi Samuel Maroele
Omphile Petunia Masibi
July Rocky Mathabanzini
Tendamudzimu Ivan Mathode
Ellen Ditaba Matsane
Lungile Mbata
Nyasha Karen Mhandire
Thembisiwe Miga
Caroline Mkhokho
Neo Hilda Mkwalase
Nondzakazi Mnqonywa
Brenda Matshidiso Modisaotsile
Patricia Pakiso Mokgetsengoane
Kegomoditswe Magdeline Molatlhegi
Thuso Andrew Molefe
Motlatsi Evelyn Molotsi
Tebogo Edwin Montwedi
Boikanyo Dinah Monyemangene
Hellen Mokopi Mooketsi
Tshplpfelo Mapula Mosito
Ireen Lesebang Mosweu
Banyana Olga Motlagomang
Funeka Nomvula Mthembu
Themba Phakathi
Mapule Ozma Phatshwane
Victor Kgothatso Rameetse
Kelebogile Magdeline Segaetsho
Ni Ni Sein
Melissa Neo Senne
Sifiso Cornelius Shezi
Zona Sithetho
Bongiwe Stofile
Mando Mmakhora Thaba
Nosisa Charity Thandeka
Lethabo Collen Theko
Dimakatso Sylvia Tsagae

Vaccine and Infectious Disease Division, Fred Hutchinson Cancer Research Center, Seattle, WA, USA.
Bhavesh Borate
Eva Chung
Michelle Chung
Ellis Hughes
Alicia Sato
Steven Self

The Cross-sectional TB Cohort Study Team

South African Tuberculosis Vaccine Initiative, Institute of Infectious Disease and Molecular Medicine, Division of Immunology, Department of Pathology, University of Cape Town, South Africa.
Hadn Africa
Janelle Botes
Fatoumatta Darboe
Elizabeth Filander
Lebohang Makhethe
Sindile Matiwane
Melissa Murphy
Constance Schreuder
Marcia Steyn
Michele van Rooyen
Noncedo Xoyana

Desmond Tutu HIV Centre, and Institute of Infectious Disease and Molecular Medicine (IDM), University of Cape Town, Cape Town, South Africa
Carl Morrow
Robin Wood

Supplementary Notes 2

Signature failure rate

Although different pass rates may have biased comparison of performance between signature, the primary aim of the study was to compare individual signature performance to the WHO Target Product Profile (TPP) criteria benchmarks rather than against each other. Hence samples with failed signatures were still included in analysis to increase statistical power. Failure rate is also an indicator of signature robustness; an important criterion when selecting biomarkers for further development and clinical translation. The high failure rate of the Suliman4 signature was attributed to the GAS6 primer-probe assay, which performed well in assay qualification (**Table S3 in Supplementary Data 1**) and validation in high yield and purity manually extracted PAXgene RNA samples. However, performance of the GAS6 assay was less robust in lower yield and quality RNA from automated robotic extraction in the CORTIS studies. Due to the pair-wise ensemble structure of three up- and three down-regulated genes, RISK6 score can still be calculated even if one or more transcript is not detected due to failed PCR amplification.[1] As a result, RISK6 was resilient to single failed PCR reactions, with highest signature pass rate. Only HIV-uninfected participants with successful measurement of the RISK11 score were enrolled in the CORTIS-01 study, whereas HIV-infected participants were enrolled irrespective of RISK11 score in CORTIS-HR. This effectively excluded participants in CORTIS-01 with very low yield or purity RNA samples, resulting in a lower signature failure rates in CORTIS-01 as compared to CORTIS-HR.

Supplementary Figures

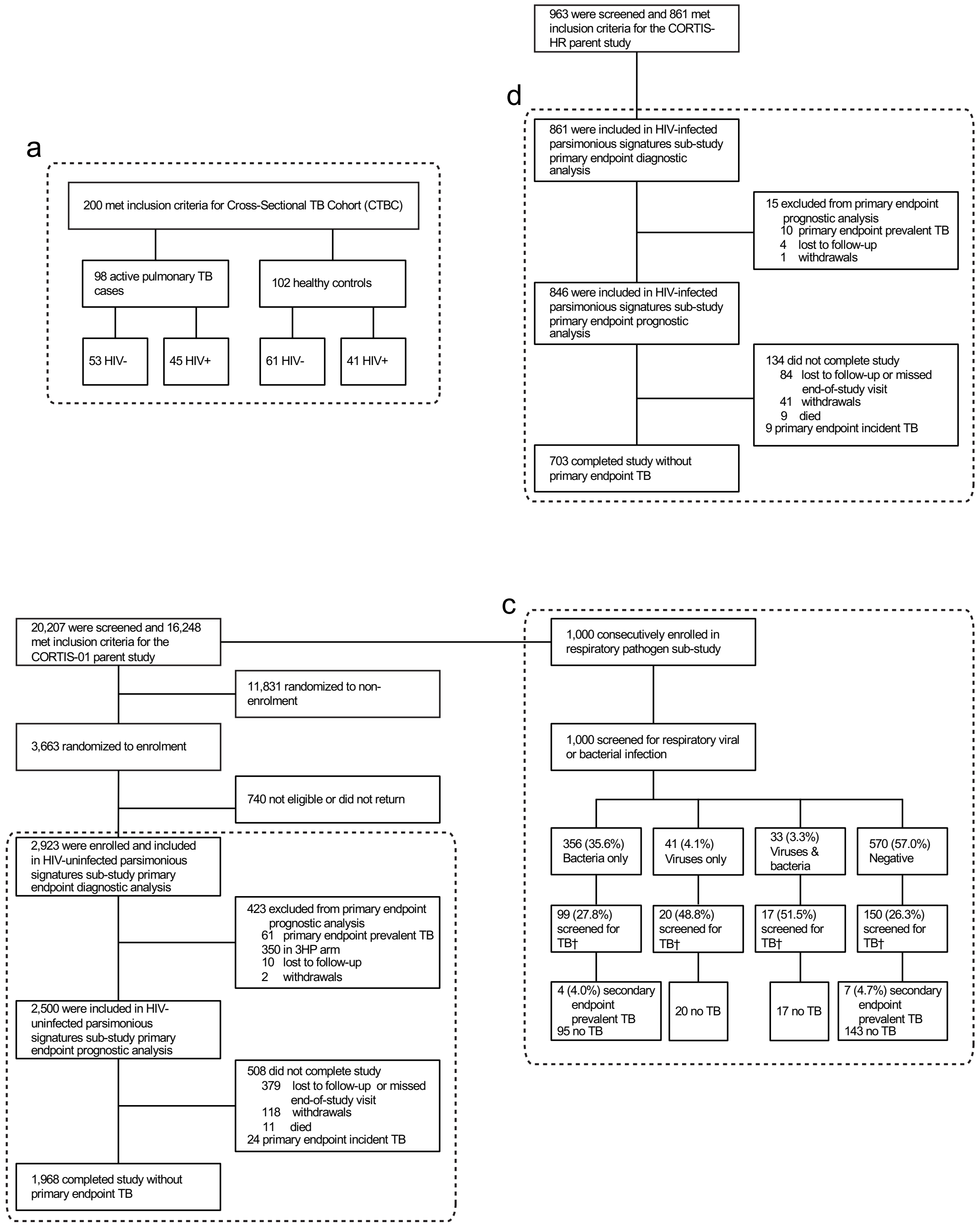

Figure S1: Study flow diagram

(a) HIV-infected and HIV-uninfected participants with and without TB were recruited into the Cross-Sectional TB Cohort (CTBC) case-control study. HIV-uninfected participants were recruited from the CORTIS-01 parent study and co-enrolled into the (b) HIV-uninfected parsimonious signatures sub-study and (c) respiratory pathobionts sub-study. HIV-infected participants were recruited from the CORTIS-HR parent study and co-enrolled into the (d) HIV-infected parsimonious signatures sub-study. For the CTBC study and respiratory pathobionts sub-study, TB disease was defined by a single sputum sample positive for *Mtb* on either Xpert MTB/RIF and/or liquid culture at enrolment. The coprimary endpoints in the CORTIS studies were baseline prevalent TB disease and incident disease through 15 months follow-up confirmed by a positive Xpert MTB/RIF, Xpert Ultra, or MGIT culture, on two or more separate sputum samples collected within any 30-day period. The secondary endpoint was microbiologically-confirmed TB disease on at least one sputum sample.

†Out of 1000 participants enrolled in the respiratory pathobionts cohort, only 286 (28.6%) participants were co-enrolled in the CORTIS-01 parent study and investigated for TB at enrolment; 11/286 (3.8%) had prevalent TB confirmed by *Mycobacterium tuberculosis* liquid culture and/or Xpert MTB/RIF.

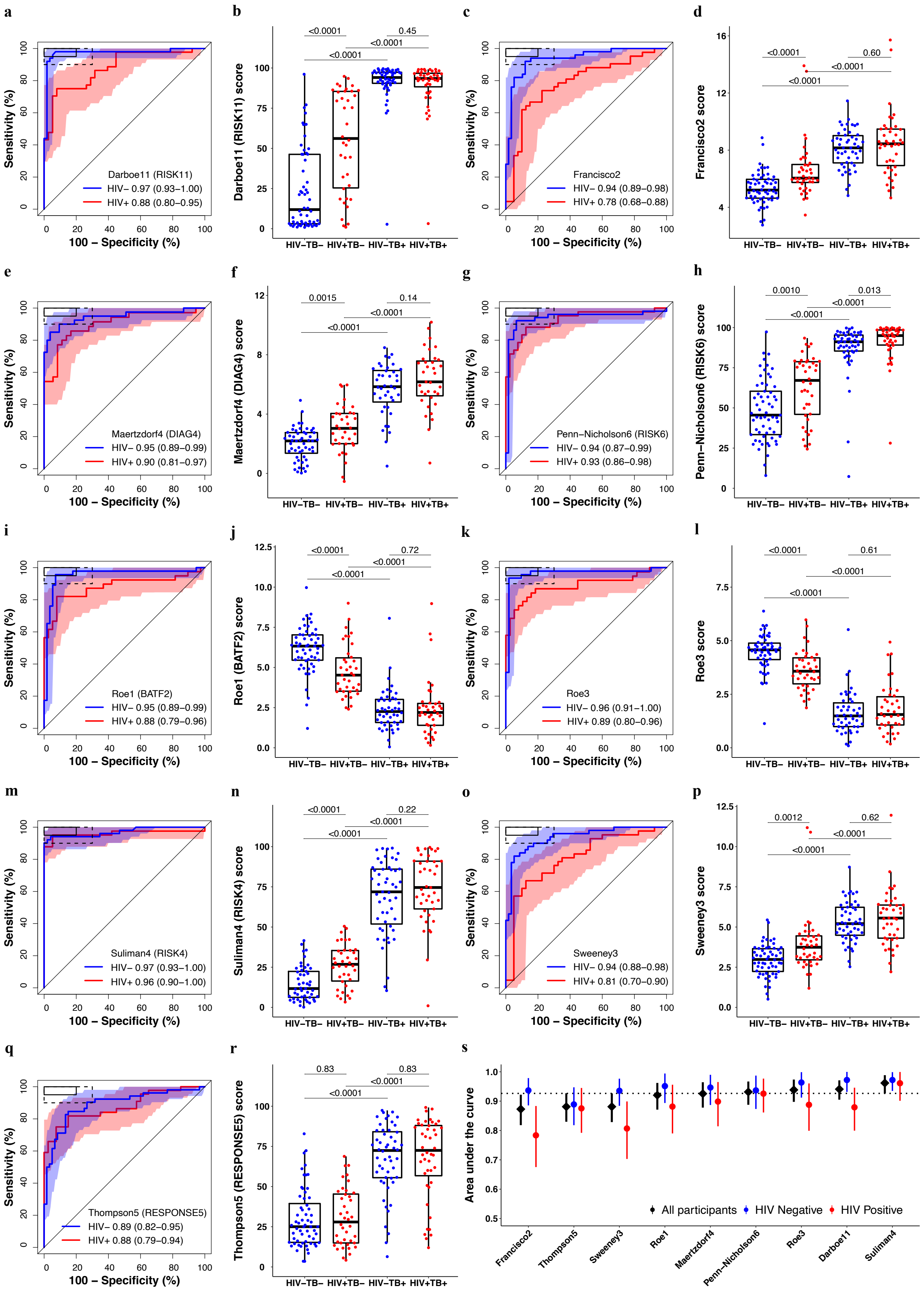
a
Sensitivity (%)
100 – Specificity (%)
Darboe11 (RISK11)
HIV− 0.97 (0.93–1.00)
HIV+ 0.88 (0.80–0.95)
b
Darboe11 (RISK11) score
<0.0001
0.45
<0.0001
<0.0001
HIV−TB− HIV+TB− HIV−TB+ HIV+TB+
c
Francisco2
HIV− 0.94 (0.89–0.98)
HIV+ 0.78 (0.68–0.88)
d
Francisco2 score
<0.0001
0.60
<0.0001
<0.0001
e
Maertzdorf4 (DIAG4)
HIV− 0.95 (0.89–0.99)
HIV+ 0.90 (0.81–0.97)
f
Maertzdorf4 (DIAG4) score
0.0015
0.14
<0.0001
<0.0001
g
Penn-Nicholson6 (RISK6)
HIV− 0.94 (0.87–0.99)
HIV+ 0.93 (0.86–0.98)
h
Penn-Nicholson6 (RISK6) score
0.0010
0.013
<0.0001
<0.0001
i
Roe1 (BATF2)
HIV− 0.95 (0.89–0.99)
HIV+ 0.88 (0.79–0.96)
j
Roe1 (BATF2) score
<0.0001
0.72
<0.0001
<0.0001
k
Roe3
HIV− 0.96 (0.91–1.00)
HIV+ 0.89 (0.80–0.96)
l
Roe3 score
<0.0001
0.61
<0.0001
<0.0001
m
Suliman4 (RISK4)
HIV− 0.97 (0.93–1.00)
HIV+ 0.96 (0.90–1.00)
n
Suliman4 (RISK4) score
<0.0001
0.22
<0.0001
<0.0001
o
Sweeney3
HIV− 0.94 (0.88–0.98)
HIV+ 0.81 (0.70–0.90)
p
Sweeney3 score
0.0012
0.62
<0.0001
<0.0001
q
Thompson5 (RESPONSE5)
HIV− 0.89 (0.82–0.95)
HIV+ 0.88 (0.79–0.94)
r
Thompson5 (RESPONSE5) score
0.83
0.83
<0.0001
<0.0001
s
Area under the curve
All participants
HIV Negative
HIV Positive
Francisco2
Thompson5
Sweeney3
Roe1
Maertzdorf4
Penn–Nicholson6
Roe3
Darboe11
Suliman4

Figure S2: Parsimonious signatures diagnostic performance and signature score distributions in the Cross-sectional Tuberculosis Cohort (CTBC) study

Paired receiver operating characteristic (ROC) curves and box-and-whisker plots for the (a-b) Darboe11 (RISK11), (c-d) Francisco2, (e-f) Maertzdorf4 (DIAG4), (g-h) Penn-Nicholson6 (RISK6), (i-j) Roe1 (BATF2), (k-l) Roe3, (m-n) Suliman4 (RISK4), (o-p) Sweeney3, and (Q-R) Thompson5 (RESPONSE5) signatures in the Cross-sectional Tuberculosis Cohort (CTBC) study.

The ROC curves depict diagnostic performance (area under the curve, AUC, with 95% CI) of the parsimonious signatures for diagnosing TB, stratified by HIV status. The shaded areas represent the 95% CIs. The solid box depicts the optimal criteria (95% sensitivity and 80% specificity) and the dashed box depicts the minimal criteria (90% sensitivity and 70% specificity) set out in the WHO Target Product Profile for a triage test.[2]

The box-and-whisker plots depict signature score distribution by HIV (HIV+/HIV-) and prevalent TB disease (TB+/TB-) status. Each dot represents a participant. p values for comparison of median signature scores between groups in box-and-whisker plots were calculated with the Mann-Whitney *U* test and corrected for multiple comparisons by use of the Benjamini-Hochberg Procedure.[3] Boxes depict the IQR, the midline represents the median, and the whiskers indicate the IQR ± (1.5 × IQR).

(S) Summary of signature diagnostic performance in order of AUC estimates in all participants. The diagnostic AUC estimates in HIV-infected and HIV-uninfected participant sub-groups are also shown. The midline indicates the AUC estimate, the error bars indicate the 95% CIs, and the black dotted line indicates the lower bound of the 95% CI for the best performing signature for all participants.

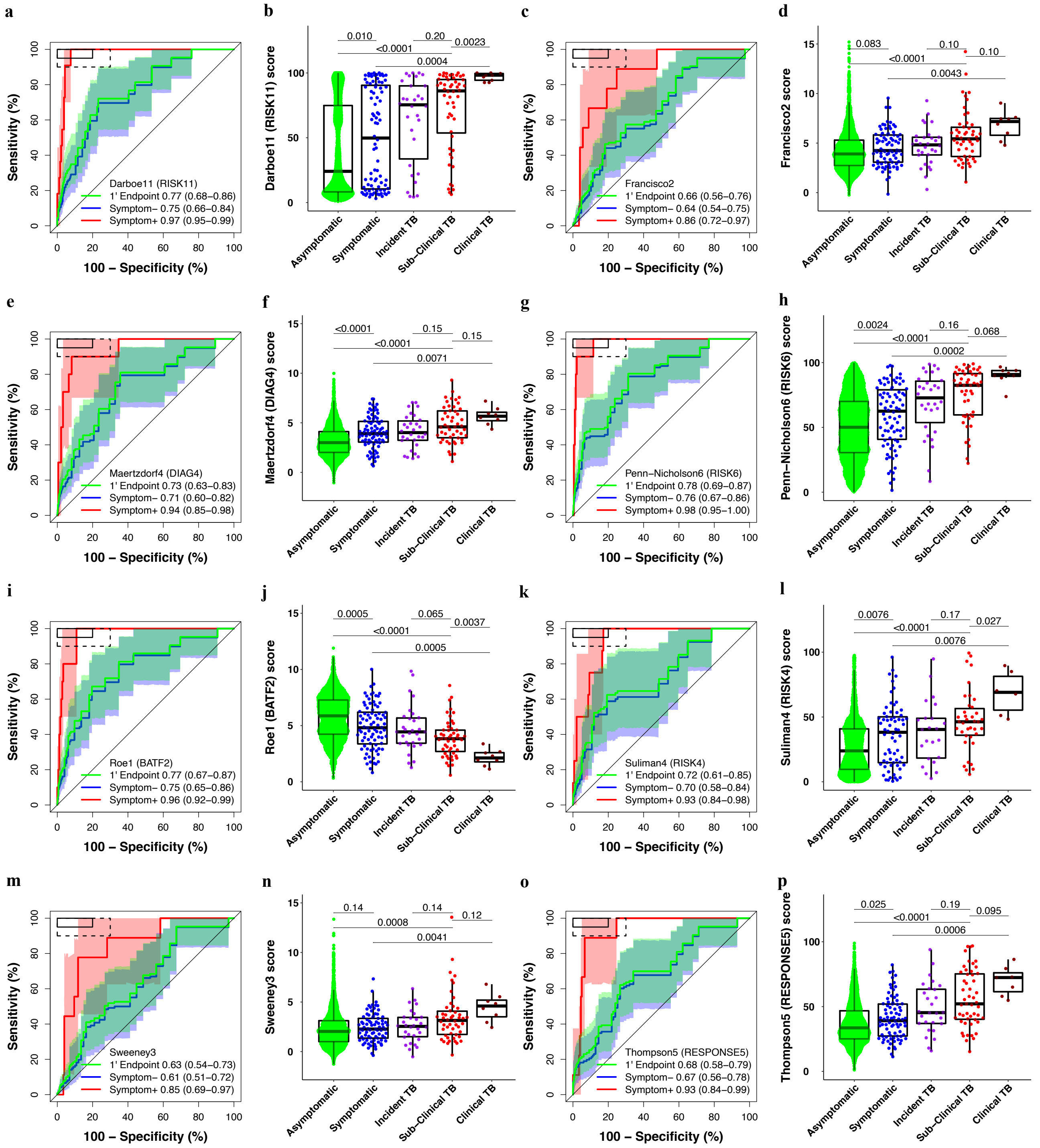

Figure S3: Parsimonious signature diagnostic performance for prevalent TB and signature score distributions in people without HIV

Paired receiver operating characteristic (ROC) curves and box-and-whisker plots for the (a-b) Darboe11 (RISK11), (c-d) Francisco2, (e-f) Maertzdorf4 (DIAG4), (g-h) Penn-Nicholson6 (RISK6), (i-j) Roe1 (BATF2), (k-l) Suliman4 (RISK4), (m-n) Sweeney3, and (o-p) Thompson5 (RESPONSE5) signatures in the CORTIS-01 study of people without HIV. The ROC curves depict diagnostic performance (area under the curve, AUC, with 95% CI) of the parsimonious signatures for the primary endpoint (1' Endpoint), i.e. TB diagnosed on two or more liquid culture-positive or Xpert MTB/RIF-positive sputum samples. The ROC curves show participants with symptomatic clinical prevalent TB versus symptomatic controls (Symptom+), and participants with asymptomatic, subclinical prevalent TB versus asymptomatic controls (Symptom–). The shaded areas represent the 95% CIs. The solid box depicts the optimal criteria (95% sensitivity and 80% specificity) and the dashed box depicts the minimal criteria (90% sensitivity and 70% specificity) set out in the WHO Target Product Profile for a triage test.[2] The box-and-whisker plots depict signature score (measured at enrolment) distribution by symptom status (each dot represents a participant) in asymptomatic and symptomatic participants with no TB, participants who progressed to incident TB, and participants with prevalent subclinical (asymptomatic) and clinical (symptomatic) TB. Prevalent and incident TB comprised all primary endpoint cases. Symptoms were recorded at the time of enrolment for participants without TB and those with prevalent TB. p values for comparison of median signature scores between groups in box-and-whisker plots were calculated with the Mann-Whitney *U* test and corrected for multiple comparisons by use of the Benjamini-Hochberg Procedure.[3] Boxes depict the IQR, the midline represents the median, and the whiskers indicate the IQR ± (1.5 × IQR).

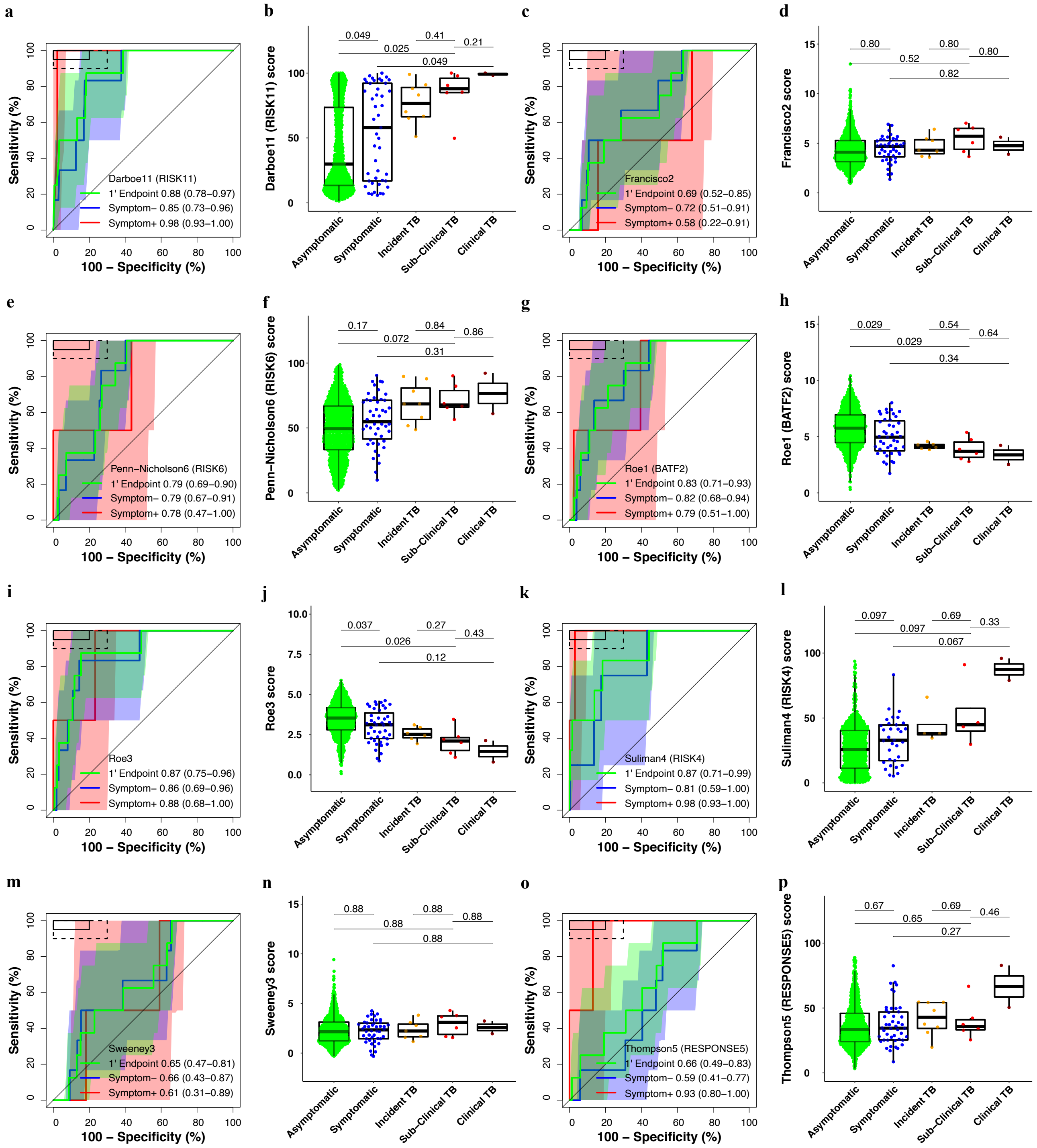

Figure S4: Parsimonious signatures diagnostic performance for prevalent TB and signature score distributions in people living with HIV. Paired receiver operating characteristic (ROC) curves and box-and-whisker plots for the (a-b) Darboe11 (RISK11), (c-d) Francisco2, (e-f) Penn-Nicholson6 (RISK6), (g-h) Roe1 (BATF2), (i-j) Roe3, (k-l) Suliman4 (RISK4), (m-n) Sweeney3, and (o-p) Thompson5 (RESPONSE5) signatures in the CORTIS-HR study of people living with HIV. The ROC curves depict diagnostic performance (area under the curve, AUC, with 95% CI) of the parsimonious signatures for TB diagnosed for the primary endpoint (1' Endpoint), i.e. TB diagnosed on two or more liquid culture-positive or Xpert MTB/RIF-positive sputum samples. The ROC curves show participants with symptomatic clinical prevalent TB versus symptomatic controls (Symptom+), and participants with asymptomatic subclinical prevalent TB versus asymptomatic controls (Symptom–). The shaded areas represent the 95% CIs. The solid box depicts the optimal criteria (95% sensitivity and 80% specificity) and the dashed box depicts the minimal criteria (90% sensitivity and 70% specificity) set out in the WHO Target Product Profile for a triage test.[2] The box-and-whisker plots depict signature score (measured at enrolment) distribution by symptom status (each dot represents a participant) in asymptomatic and symptomatic participants with no TB, participants who progressed to incident TB, and participants with prevalent subclinical (asymptomatic) and clinical (symptomatic) TB. Prevalent and incident TB comprised all primary endpoint cases. Symptoms were recorded at the time of enrolment for participants without TB and those with prevalent TB. p values for comparison of median signature scores between groups in box-and-whisker plots were calculated with the Mann-Whitney *U* test and corrected for multiple comparisons by use of the Benjamini-Hochberg Procedure.[3] Boxes depict the IQR, the midline represents the median, and the whiskers indicate the IQR ± (1.5 × IQR).

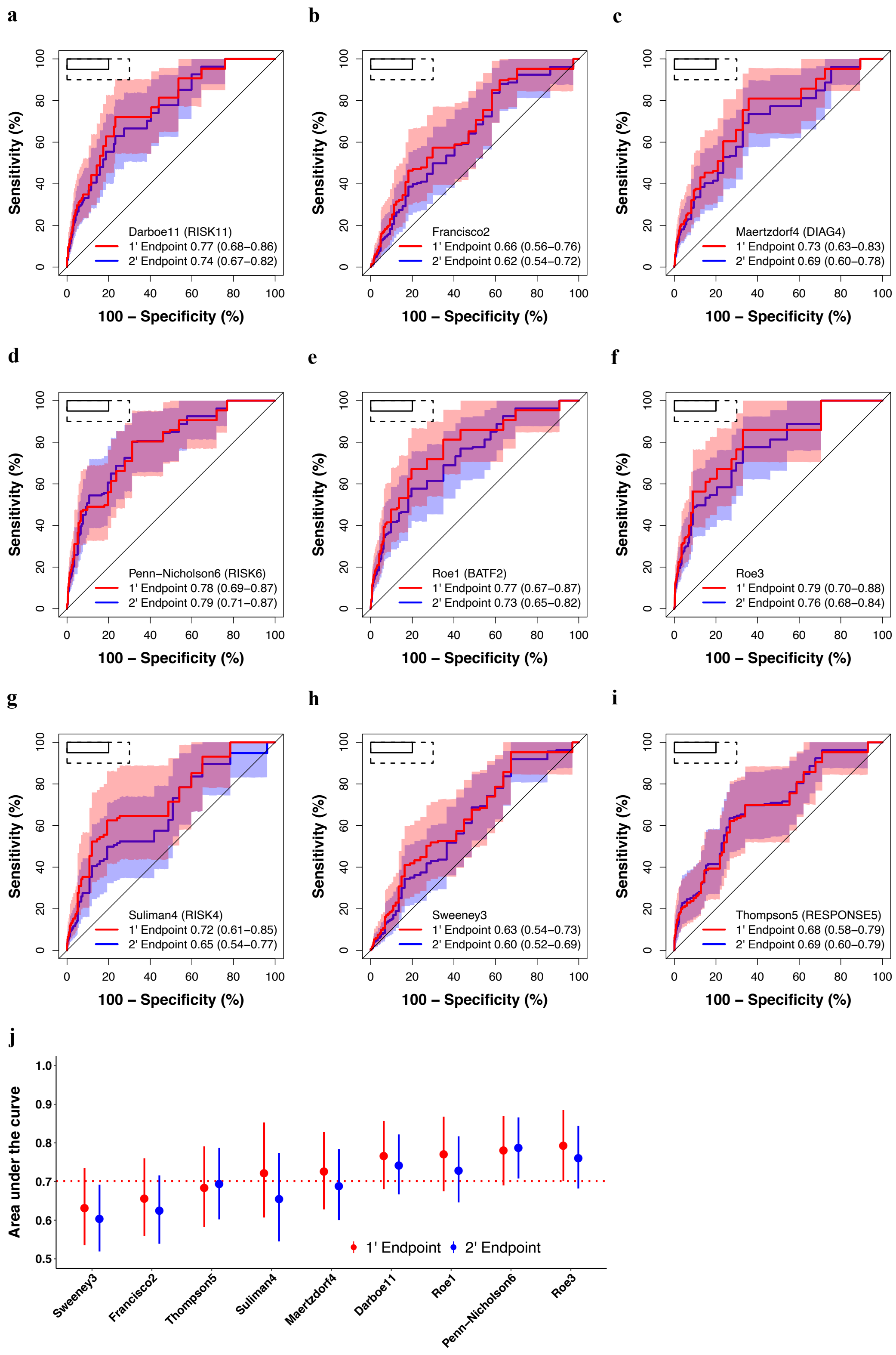

Figure S5: Parsimonious signatures diagnostic performance for primary versus secondary endpoint TB in people without HIV

Receiver operating characteristic (ROC) curves depicting primary (‘1) versus secondary (‘2) endpoint diagnostic performance (area under the curve, AUC, with 95% CI) of the (a) Darboe11 (RISK11), (b) Francisco2, (c) Maertzdorf4 (DIAG4), (d) Penn-Nicholson6 (RISK6), (e) Roe1 (BATF2), (f) Roe3, (g) Suliman4 (RISK4), (h) Sweeney3, and (i) Thompson5 (RESPONSE5) signatures in the CORTIS-01 study of people without HIV. The shaded areas represent the 95% CIs. The solid box depicts the optimal criteria (95% sensitivity and 80% specificity) and the dashed box depicts the minimal criteria (90% sensitivity and 70% specificity) set out in the WHO Target Product Profile for a triage test.[2]

(j) Summary of signature diagnostic performance in order of primary endpoint AUC estimates. The diagnostic AUC estimates for the secondary endpoint are also shown. The midline indicates the AUC estimate, the error bars indicate the 95% CIs, and the red dotted line indicates the lower bound of the 95% CI for the best performing signature for the primary endpoint.

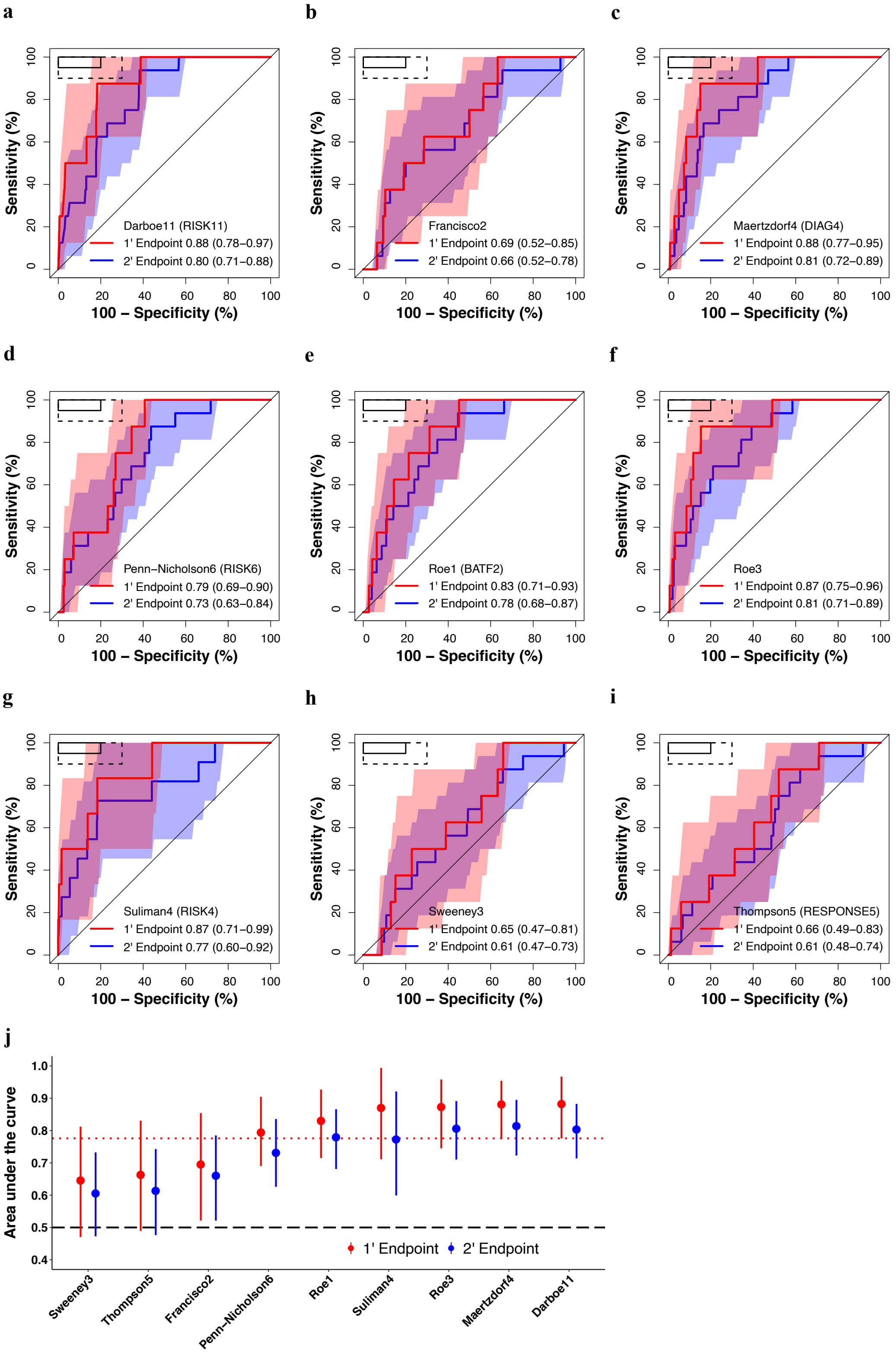

Figure S6: Parsimonious signatures diagnostic performance for primary versus secondary endpoint TB in people living with HIV

Receiver operating characteristic (ROC) curves depicting primary (‘1) versus secondary (‘2) endpoint diagnostic performance (area under the curve, AUC, with 95% CI) of the (a) Darboe11 (RISK11), (b) Francisco2, (c) Maertzdorf4 (DIAG4), (d) Penn-Nicholson6 (RISK6), (e) Roe1 (BATF2), (f) Roe3, (g) Suliman4 (RISK4), (h) Sweeney3, and (i) Thompson5 (RESPONSE5) signatures in the CORTIS-HR study of people living with HIV. The shaded areas represent the 95% CIs. The solid box depicts the optimal criteria (95% sensitivity and 80% specificity) and the dashed box depicts the minimal criteria (90% sensitivity and 70% specificity) set out in the WHO Target Product Profile for a triage test.[2]

(j) Summary of signature diagnostic performance in order of primary endpoint AUC estimates. The diagnostic AUC estimates for the secondary endpoint are also shown. The midline indicates the AUC estimate, the error bars indicate the 95% CIs, and the red dotted line indicates the lower bound of the 95% CI for the best performing signature for the primary endpoint. The black dashed line indicates an AUC cut-off of 0.5.

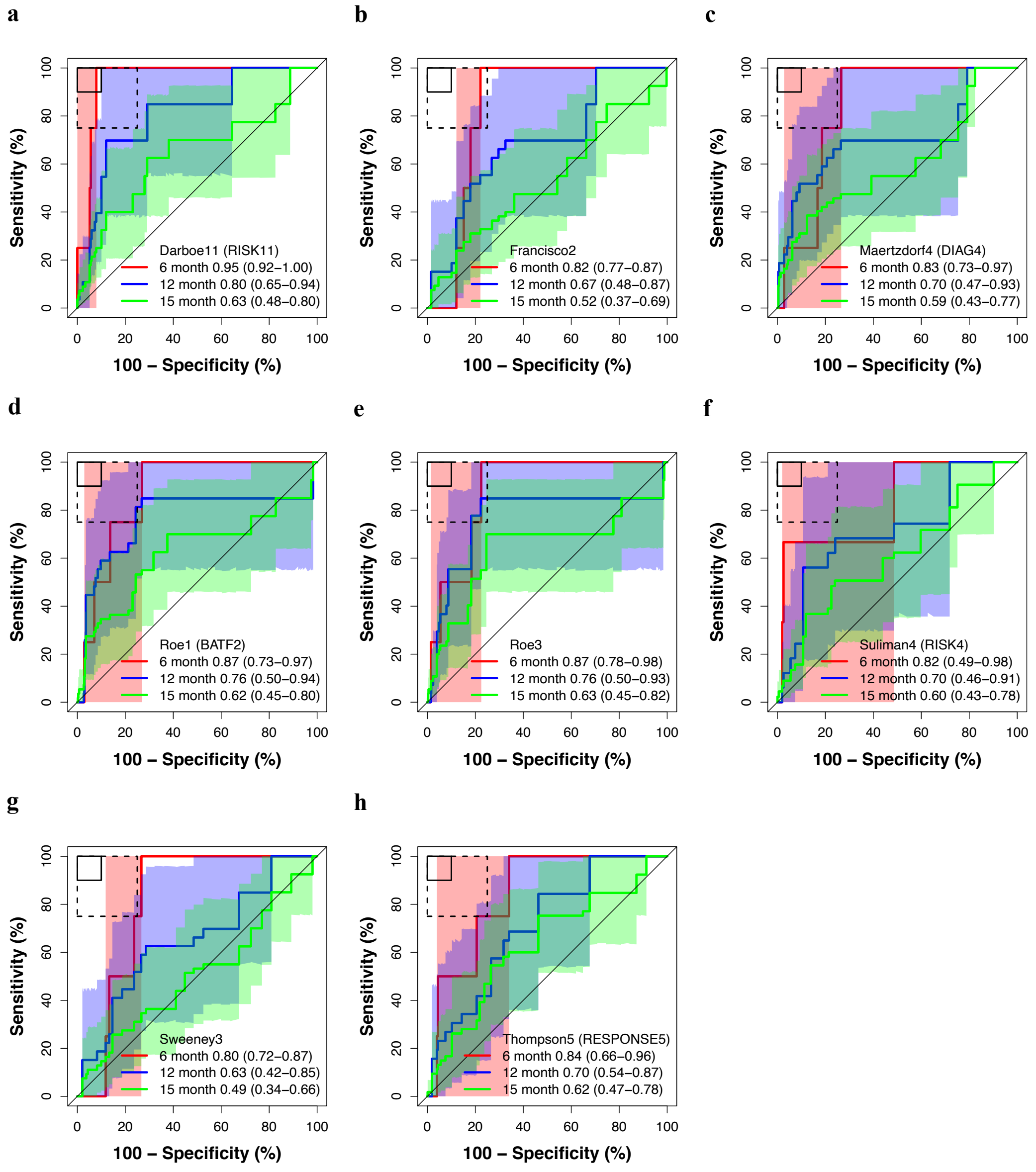

Figure S7: Parsimonious signature prognostic performance for incident TB in people without HIV

Receiver operating characteristic curves depicting prognostic performance (area under the curve, AUC, with 95% CI) of the (a) Darboe11 (RISK11), (b) Francisco2, (c) Maertzdorf4 (DIAG4), (d) Roe1 (BATF2), (e) Roe3, (f) Suliman4 (RISK4), (g) Sweeney3, and (h) Thompson5 (RESPONSE5) signatures for incident TB diagnosed on two or more liquid culture-positive or Xpert MTB/RIF-positive sputum samples (primary endpoint) through 6, 12, and 15 months follow-up in the CORTIS-01 study of people without HIV.

The shaded areas represent the 95% CIs. The solid box depicts the optimal criteria (90% sensitivity and 90% specificity) and the dashed box depicts the minimal criteria (75% sensitivity and 75% specificity) set out in the WHO Target Product Profile for an incipient TB test.[4]

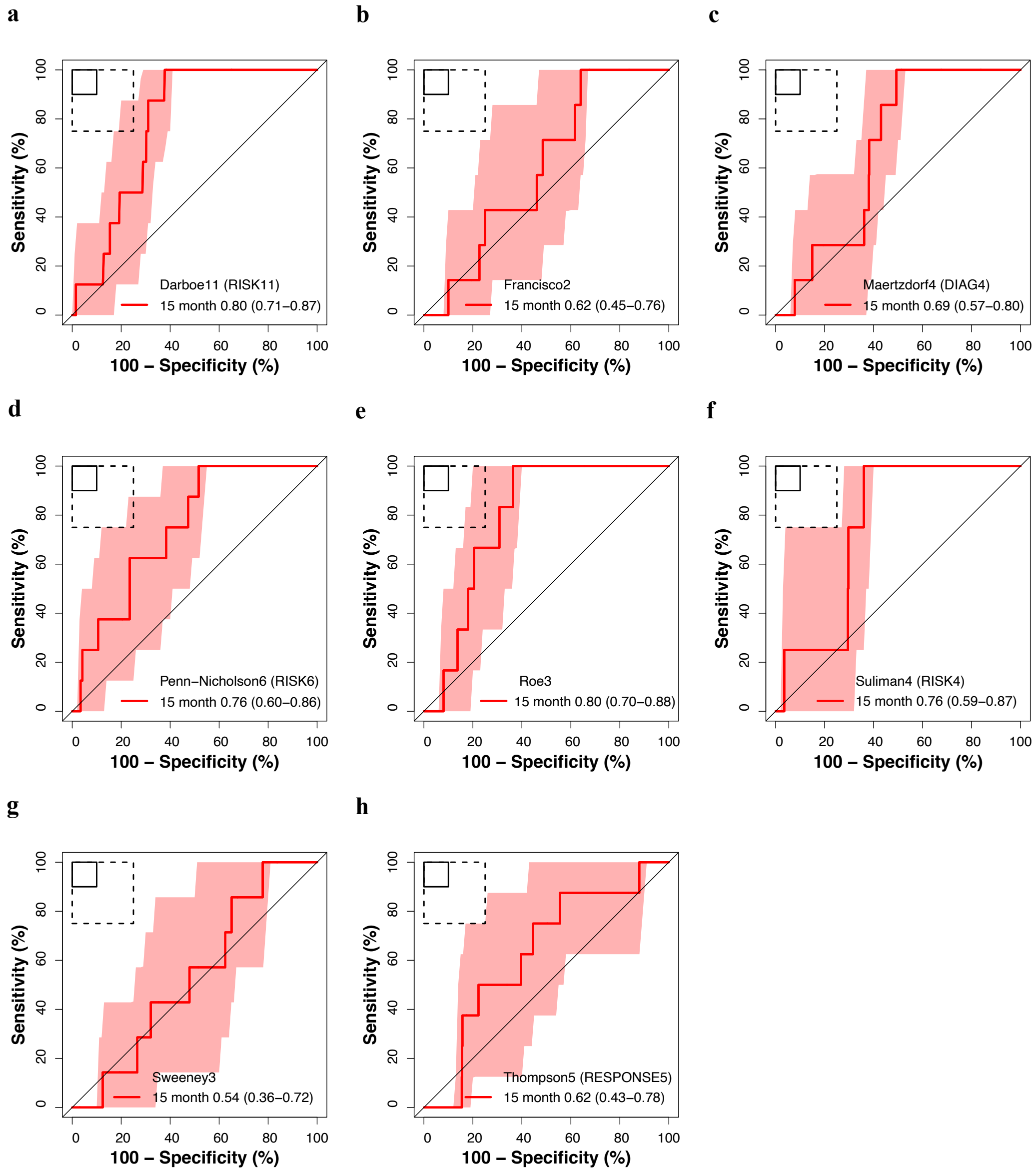

Figure S8: Parsimonious signature prognostic performance for prevalent TB in people living with HIV

Receiver operating characteristic curves depicting prognostic performance (area under the curve, AUC, with 95% CI) of the (a) Darboe11 (RISK11), (b) Francisco2, (c) Maertzdorf4 (DIAG4), (d) Penn-Nicholson6 (RISK6), (e) Roe3, (f) Suliman4 (RISK4), (g) Sweeney3, and (h) Thompson5 (RESPONSE5) signatures for incident TB diagnosed on two or more liquid culture-positive or Xpert MTB/RIF-positive sputum samples (primary endpoint) through 15 months follow-up in the CORTIS-HR study of people living with HIV.

The shaded areas represent the 95% CIs. The solid box depicts the optimal criteria (90% sensitivity and 90% specificity) and the dashed box depicts the minimal criteria (75% sensitivity and 75% specificity) set out in the WHO Target Product Profile for an incipient TB test.[4]

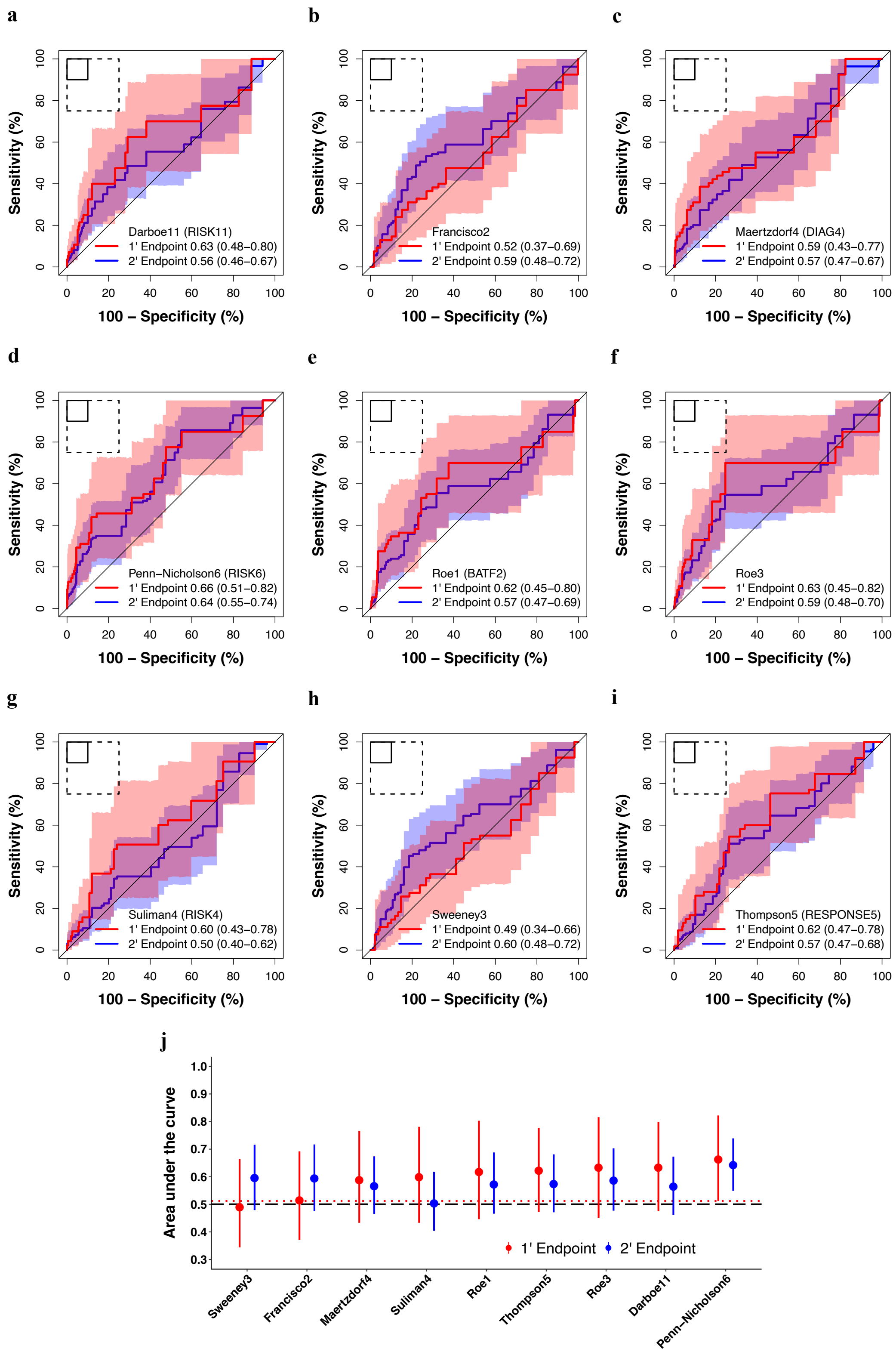

Figure S9: Parsimonious signatures prognostic performance for primary versus secondary endpoint TB through 15-months follow-up in people without HIV

Receiver operating characteristic (ROC) curves depicting primary (‘1) versus secondary (‘2) endpoint prognostic performance (area under the curve, AUC, with 95% CI) of the (a) Darboe11 (RISK11), (b) Francisco2, (c) Maertzdorf4 (DIAG4), (d) Penn-Nicholson6 (RISK6), (e) Roe1 (BATF2), (f) Roe3, (g) Suliman4 (RISK4), (h) Sweeney3, and (i) Thompson5 (RESPONSE5) signatures through 15-months follow-up in the CORTIS-01 study of people without HIV. The shaded areas represent the 95% CIs. The solid box depicts the optimal criteria (90% sensitivity and 90% specificity) and the dashed box depicts the minimal criteria (75% sensitivity and 75% specificity) set out in the WHO Target Product Profile for an incipient TB test.[4]

(j) Summary of signature prognostic performance in order of primary endpoint AUC estimates. The prognostic AUC estimates for the secondary endpoint are also shown. The midline indicates the AUC estimate, the error bars indicate the 95% CIs, the red dotted line indicates the lower bound of the 95% CI for the best performing signature for the primary endpoint, and the black dashed line indicates an AUC cut-off of 0.5.

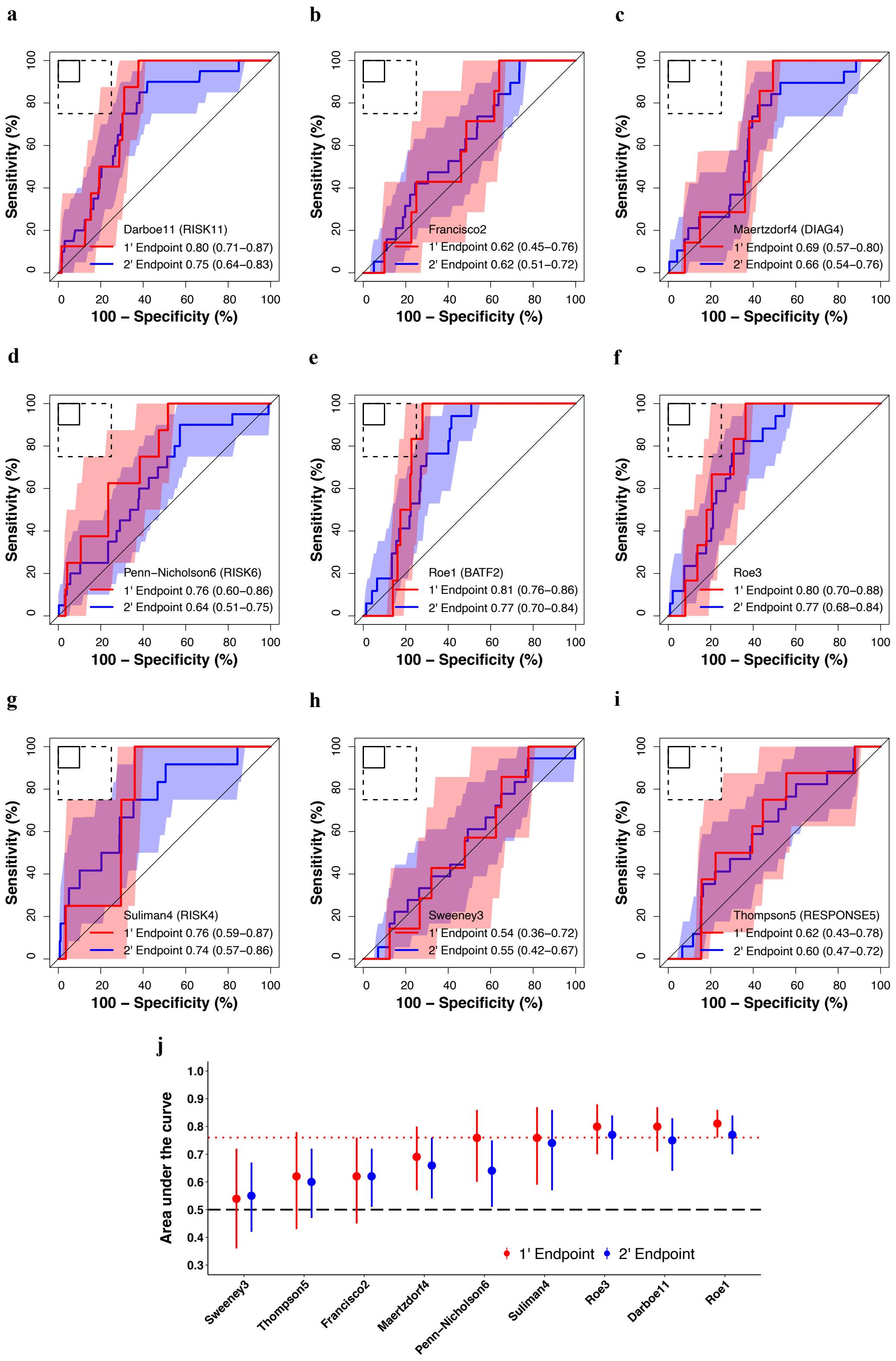

Figure S10: Parsimonious signatures prognostic performance for primary versus secondary endpoint TB through 15-months follow-up in people living with HIV

Receiver operating characteristic (ROC) curves depicting primary ('1) versus secondary ('2) endpoint prognostic performance (area under the curve, AUC, with 95% CI) of the (a) Darboe11 (RISK11), (b) Francisco2, (c) Maertzdorf4 (DIAG4), (d) Penn-Nicholson6 (RISK6), (e) Roe1 (BATF2), (f) Roe3, (g) Suliman4 (RISK4), (h) Sweeney3, and (i) Thompson5 (RESPONSE5) signatures through 15-months follow-up in the CORTIS-HR study of people living with HIV. The shaded areas represent the 95% CIs. The solid box depicts the optimal criteria (90% sensitivity and 90% specificity) and the dashed box depicts the minimal criteria (75% sensitivity and 75% specificity) set out in the WHO Target Product Profile for an incipient TB test.[4] (j) Summary of signature prognostic performance in order of primary endpoint AUC estimates. The prognostic AUC estimates for the secondary endpoint are also shown. The midline indicates the AUC estimate, the error bars indicate the 95% CIs, the red dotted line indicates the lower bound of the 95% CI for the best performing signature for the primary endpoint, and the black dashed line indicates an AUC cut-off of 0.5.

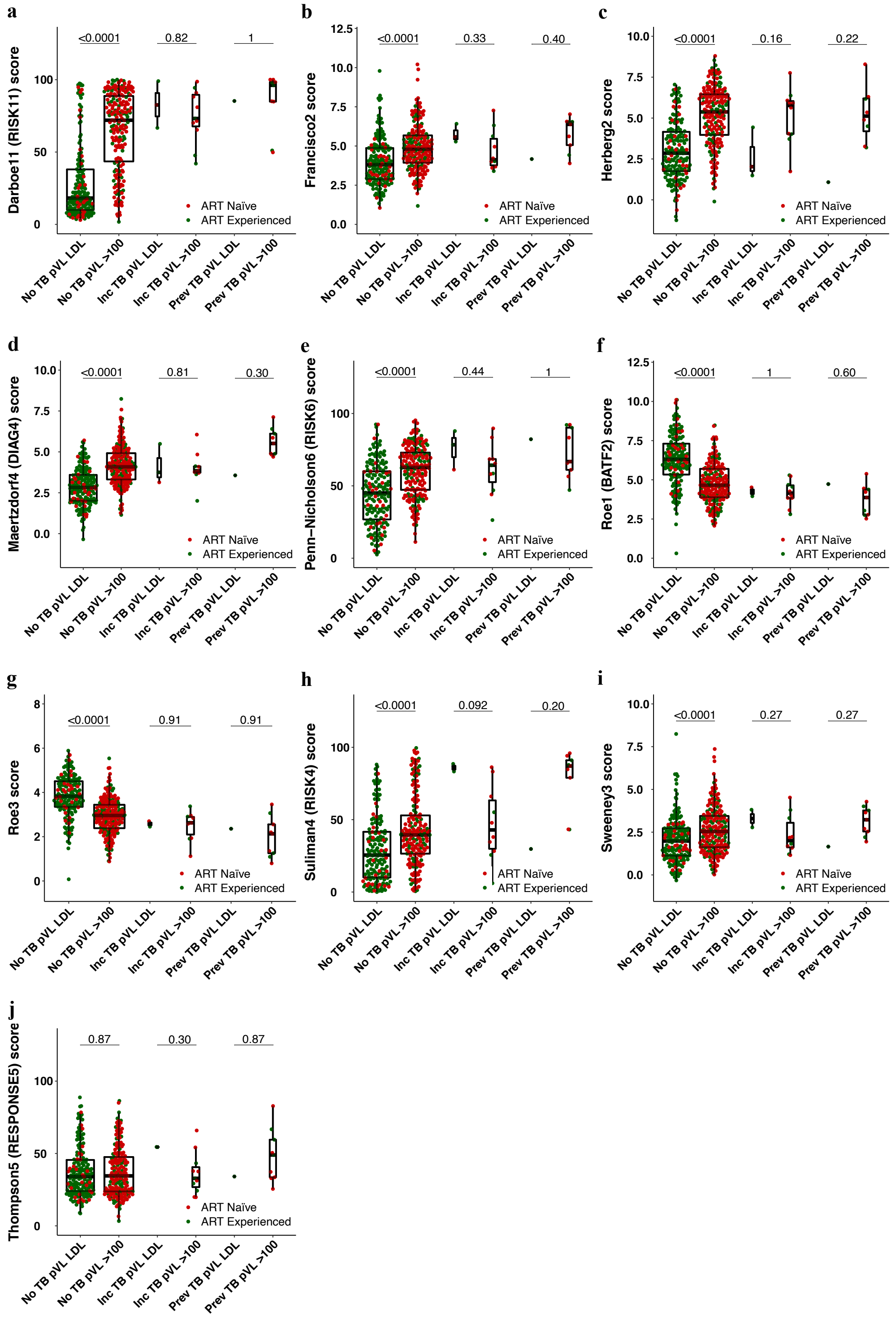

Figure S11: Parsimonious signatures score distribution by HIV plasma viral load

Box-and-whisker plots depicting distributions of the (a) Darboe11 (RISK11), (b) Francisco2, (c) Herberg2, (d) Maertzdorf4 (DIAG4), (e) Penn-Nicholson6 (RISK6), (f) Roe1 (BATF2), (g) Roe3, (h) Suliman4 (RISK4), (i) Sweeney3, and (j) Thompson5 (RESPONSE5) signature scores measured at baseline in the CORTIS-HR study stratified by HIV plasma viral load (copies per mL) and TB status. Prevalent (Prev) and incident (Inc) TB comprised all microbiologically confirmed secondary endpoint cases (i.e. TB confirmed on at least one sputum sample). HIV plasma viral load (pVL) is stratified into two groups: lower than the detectable limit (LDL) of 100 copies per mL and greater than 100 copies per mL (>100).
Each dot represents a participant. p values for comparison of median signature scores between groups in box-and-whisker plots were calculated with the Mann-Whitney *U* test and corrected for multiple comparisons by use of the Benjamini-Hochberg Procedure.[3] Boxes depict the IQR, the midline represents the median, and the whiskers indicate the IQR ± (1.5 × IQR).

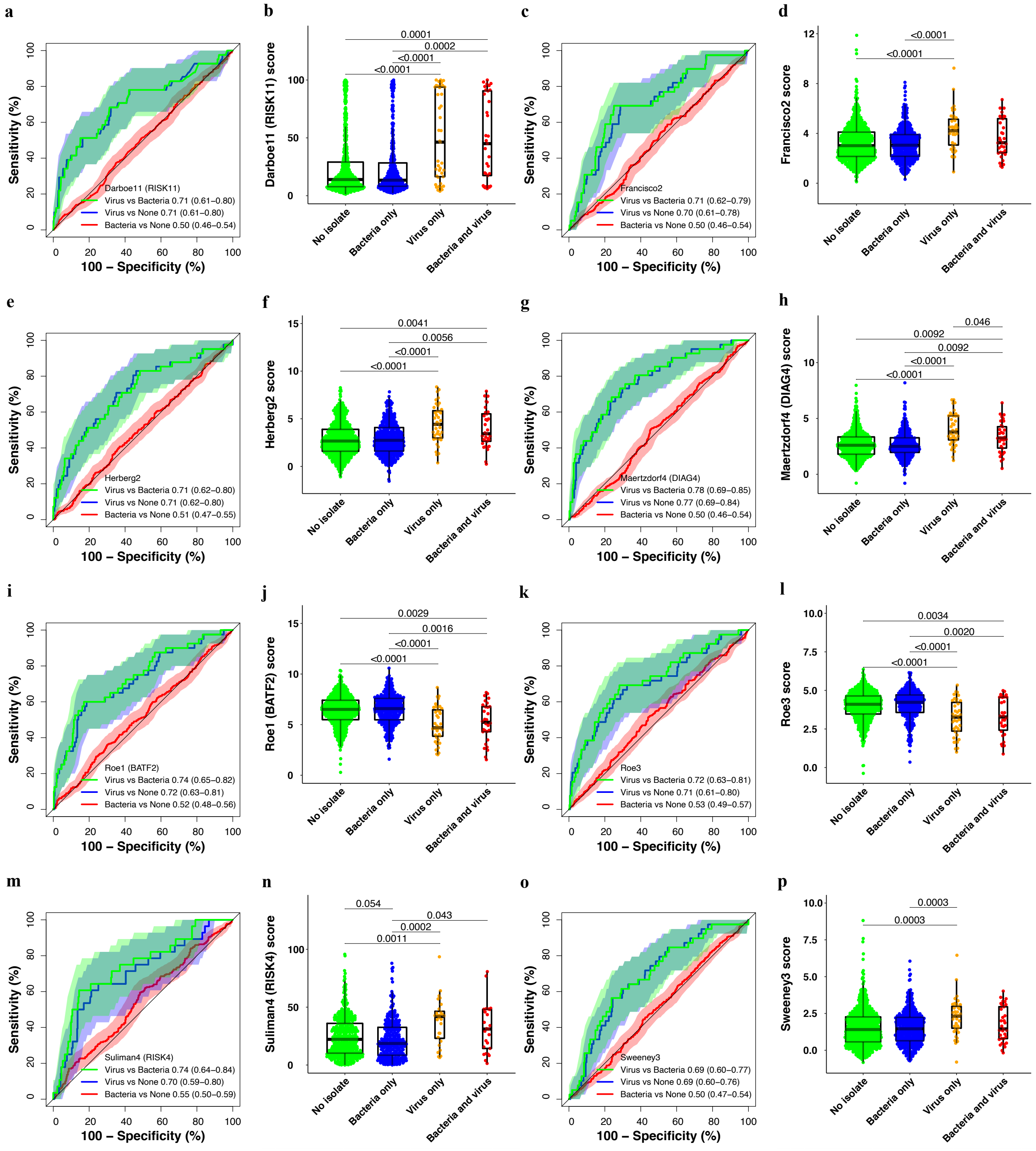

Figure S12: Parsimonious signature performance for differentiating participants with viral or bacterial upper respiratory tract pathobionts, and those without any pathobionts

Paired receiver operating characteristic (ROC) curves and box-and-whisker plots for the (a-b) Darboe11 (RISK11), (c-d) Francisco2, (e-f) Herberg2, (g-h) Maertzdorf4 (DIAG4), (i-j) Roe1 (BATF2), (k-l) Roe3, (m-n) Suliman4 (RISK4), and (o-p) Sweeney3 signatures in all participants without prevalent TB in the respiratory pathobionts sub-study (11 participants with secondary endpoint prevalent TB excluded). The box-and-whisker plots depict signature score distributions in participants with no upper respiratory pathobionts (n=563), bacterial upper respiratory pathobionts only (n=352), viral upper respiratory pathobionts only (n=41), both viral and bacterial upper respiratory pathobionts (n=33). p values for comparison of median signature scores between groups in box-and-whisker plots were calculated with the Mann-Whitney *U* test and corrected for multiple comparisons by use of the Benjamini-Hochberg Procedure.[3] Only p-values below 0.1 are shown. Each dot represents a participant. Boxes depict the IQR, the midline represents the median, and the whiskers indicate the IQR ± (1.5 × IQR). The ROC curves depict performance (area under the curve, AUC, with 95% CI) of the parsimonious signatures in differentiating between participants with viral upper respiratory pathobionts and participants with bacterial upper respiratory pathobionts, between participants with viral pathobionts and those with no pathobionts, or between participants with bacterial pathobionts and those with no pathobionts. The shaded areas represent the 95% CIs.

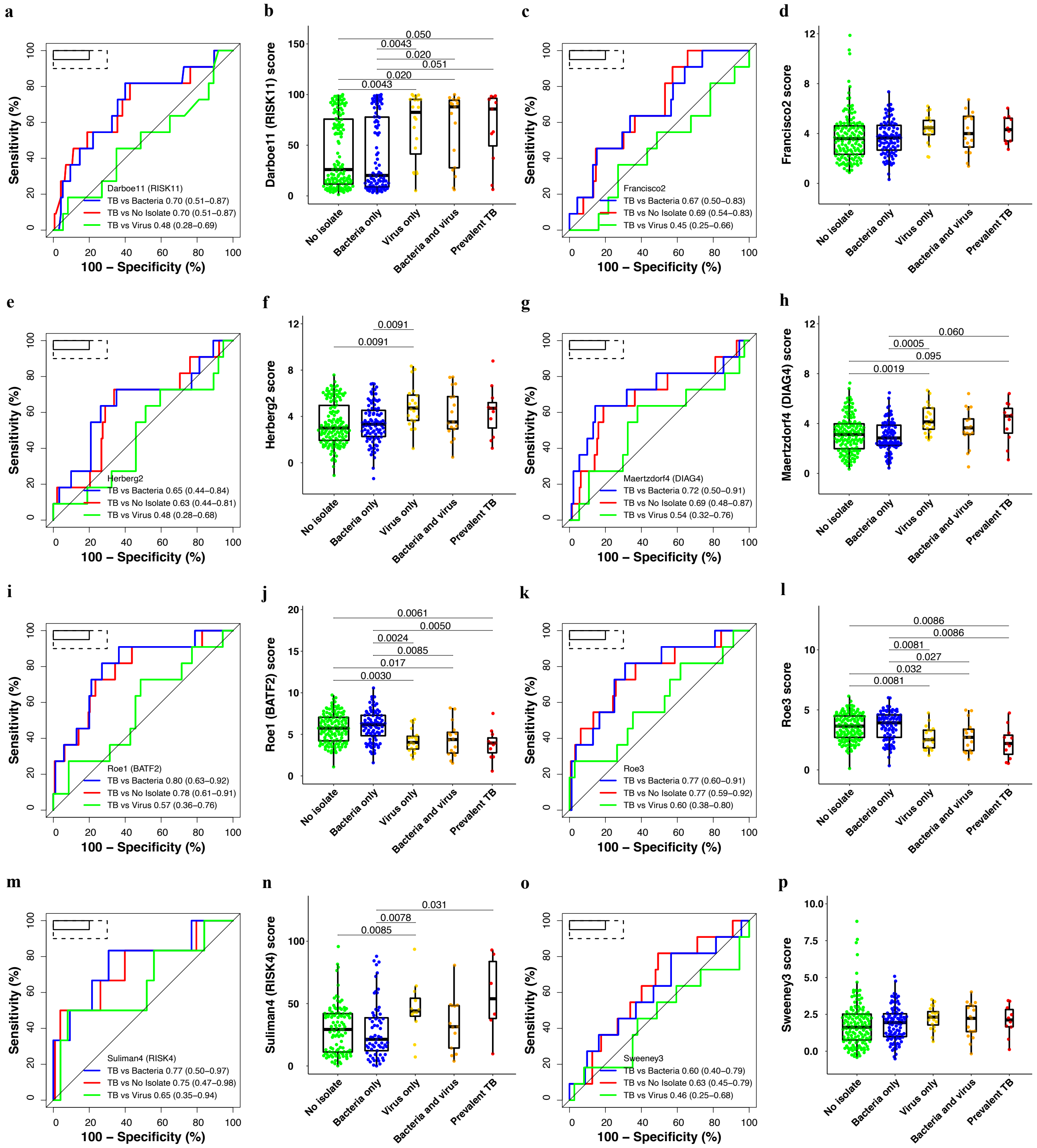

Figure S13: Parsimonious signatures performance for differentiating participants with TB from those viral or bacterial upper respiratory tract pathobionts, and those without any pathobionts

Paired receiver operating characteristic (ROC) curves and box-and-whisker plots for the (a-b) Darboe11 (RISK11), (c-d) Francisco2, (e-f) Herberg2, (g-h) Maertzdorf4 (DIAG4), (i-j) Roe1 (BATF2), (k-l) Roe3, (m-n) Suliman4 (RISK4), and (o-p) Sweeney3 signatures in all participants randomised in the CORTIS-01 study and co-enrolled in the respiratory pathobionts sub-study. Participants who were not randomised in the CORTIS-01 study, and thus not investigated for TB, are not included.

The box-and-whisker plots depict signature score distribution in participants with no upper respiratory pathobionts (n=143), bacterial upper respiratory pathobionts only (n=95), viral upper respiratory pathobionts only (n=20), both viral and bacterial upper respiratory pathobionts (n=17), and *Mycobacterium tuberculosis* (n=11) detected on GeneXpert MTB/RIF or MGIT culture (microbiologically-confirmed secondary endpoint prevalent TB; i.e. TB confirmed on at least one sputum sample). p values for comparison of median signature scores between groups in box-and-whisker plots were calculated with the Mann-Whitney *U* test and corrected for multiple comparisons by use of the Benjamini-Hochberg Procedure.[3] Only p-values below 0.1 are shown. Each dot represents a participant. Boxes depict the IQR, the midline represents the median, and the whiskers indicate the IQR ± (1.5 × IQR).

The ROC curves depict performance (area under the curve, AUC, with 95% CI) of the parsimonious signatures in differentiating between participants with prevalent TB (n=11) and participants with viral upper respiratory pathobionts (n=37), participants with bacterial upper respiratory pathobionts only (n=95), and participants with no pathobionts (n=143). Participants with both viral and bacterial pathobionts (n=17) were included in the group with viral pathobionts only (n=20) as the presence of bacterial pathobionts did not appear to affect signature scores.

The solid box depicts the optimal criteria (95% sensitivity and 80% specificity) and the dashed box depicts the minimal criteria (90% sensitivity and 70% specificity) set out in the WHO Target Product Profile for a triage test.[2]

Supplementary References

1. Penn-Nicholson A, Mbandi SK, Thompson E, Mendelsohn SC, Suliman S, Chegou NN, et al. RISK6, a 6-gene transcriptomic signature of TB disease risk, diagnosis and treatment response. Sci Rep. 2020;10(1):8629. doi: 10.1038/s41598-020-65043-8.
2. WHO. High-priority target product profiles for new tuberculosis diagnostics: report of a consensus meeting. Geneva: World Health Organization, 2014. https://apps.who.int/iris/handle/10665/135617 (accessed May 22, 2020).
3. Benjamini Y, Hochberg Y. Controlling the False Discovery Rate: A Practical and Powerful Approach to Multiple Testing. Journal of the Royal statistical society: series B (Methodological). 1995;57(1):289-300. doi: 10.1111/j.2517-6161.1995.tb02031.x.
4. WHO. Consensus Meeting Report: Development of a Target Product Profile (TPP) and a framework for evaluation for a test for predicting progression from tuberculosis infection to active disease. Geneva: World Health Organization, 2017. http://apps.who.int/iris/handle/10665/259176 (accessed May 22, 2020).
