# Supplementary material for: Prospective multicentre head-to-head validation of host blood transcriptomic biomarkers for pulmonary tuberculosis by real-time PCR
Source: Commun Med (Lond). 2022 Mar 10;2:26. doi: 10.1038/s43856-022-00086-8 (PMC8954216; doi:10.1038/s43856-022-00086-8)
Supplement: Supplementary file 3 — Description of Additional Supplementary Files [file 43856_2022_86_MOESM3_ESM.pdf]

Description of Additional Supplementary Files

File Name: Supplementary Data 1

Description: **Supplementary Data** contains the following **Supplementary Tables** in a single Excel (.xlsx) file.

Table S1. Parsimonious transcriptomic signatures included in panel and signature score calculation

Table S2. TaqMan PCR primer-probe panel for Darboe11 (RISK11) Fluidigm 96.96 gene expression integrated fluidic circuit

Table S3. TaqMan PCR primer-probe panel for Fluidigm 192.24 gene expression integrated fluidic circuit and assay qualification

Table S4. Penn-Nicholson6 (RISK6) signature score calculation

Table S5. Suliman4 (RISK4) signature score calculation

Table S6. Thompson5 (RESPONSE5) signature score calculation

Table S7. Diagnostic performance of transcriptomic signatures in CTBC cohort

Table S8. Baseline characteristics of enrolled CORTIS-01 study cohort and tuberculosis endpoints

Table S9. Baseline characteristics of enrolled CORTIS-HR study cohort and tuberculosis endpoints

Table S10. Primary endpoint diagnostic performance of transcriptomic signatures in CORTIS-01 cohort

Table S11. Primary endpoint diagnostic performance of transcriptomic signatures in CORTIS-HR cohort

Table S12. Primary endpoint prognostic performance of transcriptomic signatures in CORTIS-01 cohort

Table S13. Primary endpoint prognostic performance of transcriptomic signatures in CORTIS-HR cohort

Table S14. Baseline characteristics of screened CORTIS-01 study participants enrolled in the respiratory pathobionts sub-study

Table S15. CTBC cohort signature scores and metadata

Table S16. CORTIS-01 sub-study cohort signature scores and metadata

Table S17. CORTIS-HR sub-study cohort signature scores and metadata

Table S18. CORTIS-01 Respiratory Pathogens sub-study cohort signature scores and metadata
