# Supplementary material for: Prospective multicentre head-to-head validation of host blood transcriptomic biomarkers for pulmonary tuberculosis by real-time PCR
Source: Commun Med (Lond). 2022 Mar 10;2:26. doi: 10.1038/s43856-022-00086-8 (PMC8954216; doi:10.1038/s43856-022-00086-8)
Supplement: Supplementary file 5 — Reporting Summary [file 43856_2022_86_MOESM5_ESM.pdf]

nature research

Corresponding author(s): Thomas J Scriba

Last updated by author(s): Jan 18, 2022

Reporting Summary

Nature Research wishes to improve the reproducibility of the work that we publish. This form provides structure for consistency and transparency in reporting. For further information on Nature Research policies, see our Editorial Policies and the Editorial Policy Checklist.

Statistics

For all statistical analyses, confirm that the following items are present in the figure legend, table legend, main text, or Methods section.

n/a	Confirmed	
☐	☒	The exact sample size (*n*) for each experimental group/condition, given as a discrete number and unit of measurement
☒	☐	A statement on whether measurements were taken from distinct samples or whether the same sample was measured repeatedly
☐	☒	The statistical test(s) used AND whether they are one- or two-sided<br>*Only common tests should be described solely by name; describe more complex techniques in the Methods section.*
☒	☐	A description of all covariates tested
☐	☒	A description of any assumptions or corrections, such as tests of normality and adjustment for multiple comparisons
☐	☒	A full description of the statistical parameters including central tendency (e.g. means) or other basic estimates (e.g. regression coefficient) AND variation (e.g. standard deviation) or associated estimates of uncertainty (e.g. confidence intervals)
☐	☒	For null hypothesis testing, the test statistic (e.g. *F*, *t*, *r*) with confidence intervals, effect sizes, degrees of freedom and *P* value noted<br>*Give P values as exact values whenever suitable.*
☒	☐	For Bayesian analysis, information on the choice of priors and Markov chain Monte Carlo settings
☒	☐	For hierarchical and complex designs, identification of the appropriate level for tests and full reporting of outcomes
☒	☐	Estimates of effect sizes (e.g. Cohen's *d*, Pearson's *r*), indicating how they were calculated

Our web collection on statistics for biologists contains articles on many of the points above.

Software and code

Policy information about availability of computer code

Data collection	The locked-down R code used to analyse the Fluidigm 192.24 gene expression chips, with quality control filters that assessed the integrity and reproducibility of each chip, is available at bitbucket.org/satvi/sixs. See further details in Methods.
Data analysis	All statistical analyses were done in R (Boston, MA, USA), version 3.6.1. Binary ROC analysis was performed using the pROC package. Prognostic performance metrics through 15 months follow-up in CORTIS-HR were calculated by use of non-parametric methods for time-dependent ROC curve analysis from survival data using the survAM.estimate function in the survAccuracyMeasures package. Sensitivity, specificity, positive predictive value (PPV), and negative predictive value (NPV) at each threshold were calculated using binary endpoint indicators and standard formulae. The 95% CIs on diagnostic and prognostic performance estimates were calculated with a non-parametric percentile bootstrap with 10,000 resamples using the boot or pROC packages. Further details in Methods.

For manuscripts utilizing custom algorithms or software that are central to the research but not yet described in published literature, software must be made available to editors and reviewers. We strongly encourage code deposition in a community repository (e.g. GitHub). See the Nature Research guidelines for submitting code & software for further information.

Data

Policy information about availability of data

All manuscripts must include a data availability statement. This statement should provide the following information, where applicable:

- Accession codes, unique identifiers, or web links for publicly available datasets
- A list of figures that have associated raw data
- A description of any restrictions on data availability

Deidentified signature scores, clinical metadata, and TB endpoint data will be made available with publication in the supplemental material (Supplementary Tables

S15-18). The public PCR probe dataset and metadata has been deposited in Zivahub (https://doi.org/10.25375/uct.14999895), an open access data repository hosted by the University of Cape Town's institutional data repository powered by Figshare for Institutions.

Field-specific reporting

Please select the one below that is the best fit for your research. If you are not sure, read the appropriate sections before making your selection.

☒ Life sciences ☐ Behavioural & social sciences ☐ Ecological, evolutionary & environmental sciences

For a reference copy of the document with all sections, see nature.com/documents/nr-reporting-summary-flat.pdf

Life sciences study design

All studies must disclose on these points even when the disclosure is negative.

Sample size

Sample sizes for this sub-study were not pre-specified, but were based on accumulated enrolment of the CTBC and CORTIS parent studies.

Data exclusions

Participants meeting the primary prevalent TB endpoint definition, participants who did not attend further follow-up visits, and participants randomized to the RISK11-positive 3HP group (CORTIS-01 parent study only) were excluded from the primary endpoint prognostic performance analysis. This was a pre-specified exclusion.

The RISK11 signature assay panel (Supplementary Table S2) was measured in all randomised HIV-uninfected CORTIS-01 trial participants (N=2,923) and in all enrolled HIV-infected CORTIS-HR participants who had baseline RNA samples available 857/861 (99.5%).
An internal positive control sample was run on all Fluidigm gene expression chips. Chips with marked deviation in internal positive control sample primer-probe assay Ct values or RISK6 signature score from historical runs were repeated.
The parsimonious transcriptomic signature assay panel (Supplementary Table S3) was measured in 2,904/2,923 (99.3%) HIV-uninfected and 858/861 (99.7%) HIV-infected participants who had additional baseline RNA aliquots available. Samples and primer-probe assays were run in singlet, and failed signature results for individual samples were assumed to follow a random distribution, thus not repeated, and excluded from analysis. These data exclusions were pre-determined.

Replication

We did not include replication cohorts.

Randomization

Participants in the CORTIS-01 parent study were randomized to a RISK11-positive 3HP+ or 3HP- group. In this sub-study, the RISK11-positive 3HP+ group was included the primary endpoint diagnostic performance analysis at baseline (i.e. before participants received 3HP), but were excluded from the primary endpoint prognostic performance analysis. In the CORTIS-01 parent study assignment to study group based on RISK11 status was managed by an unmasked randomisation team. RISK11-positive volunteers were randomly assigned (1:2; block size 15) to either open-label 3HP (3HP-positive group), or active tuberculosis surveillance without 3HP (3HP-negative group), in accordance with a randomisation schedule generated using SAS, version 9.4. RISK11-negative volunteers were concurrently randomly assigned either to active tuberculosis surveillance (3HP-negative group) or to non-participation, to enrich the study population for RISK11-positive participants. Due to enrichment of RISK11-positive individuals in the CORTIS-01 enrolled population, analyses of signature performance required participant weighting to obtain estimates applicable to the screened population, effectively upweighting RISK11-negative participants in the CORTIS-01 analyses (i.e. inverse probability weighting).

Blinding

Transcriptomic signature scores were measured by laboratory personnel who were blinded to participant TB status. Participants and study staff responsible for TB screening were blinded to transcriptomic signature scores. The statistical analysis team had access to clinical and demographic data (including TB status), but were blinded to signature scores, to allow data cleaning and preparation of analysis scripts prior to database lock. Signature scores and TB microbiology results were maintained in different files, which were only integrated after the study database had been cleaned and locked, and group allocations unblinded in CORTIS-01.

Reporting for specific materials, systems and methods

We require information from authors about some types of materials, experimental systems and methods used in many studies. Here, indicate whether each material, system or method listed is relevant to your study. If you are not sure if a list item applies to your research, read the appropriate section before selecting a response.

Materials & experimental systems

n/a	Involved in the study	
☒	☐	Antibodies
☒	☐	Eukaryotic cell lines
☒	☐	Palaeontology and archaeology
☒	☐	Animals and other organisms
☐	☒	Human research participants
☒	☐	Clinical data
☒	☐	Dual use research of concern

Methods

n/a	Involved in the study	
☒	☐	ChIP-seq
☒	☐	Flow cytometry
☒	☐	MRI-based neuroimaging

Human research participants

Policy information about studies involving human research participants

Population characteristics	Population characteristics are detailed in Supplementary Tables S8, S9, and S14.
Recruitment	Briefly, healthy adult volunteers without clinical suspicion of TB, residing in five TB endemic communities in South Africa (Durban, Klerksdorp, Ravensmead, Rustenburg, and Worcester), were recruited through word-of-mouth, house-to-house visits, and liaison with non-governmental organisations. Recruitment did not target symptomatic individuals seeking health care or other high-risk groups. Eligible participants aged 18–59 years were without comorbidities (except for HIV) and did not have known TB disease, or household exposure to individuals with multi-drug resistant TB, within the prior three years.
Ethics oversight	The study protocol was approved by the University of Cape Town Faculty of Health Sciences Human Research Ethics Committee (HREC 812/2017), and institutional human research ethics committees at each participating site.

Note that full information on the approval of the study protocol must also be provided in the manuscript.
